# Selective cellulose fibril release from hardwoods libriform tissue

**DOI:** 10.1038/s42004-026-02094-4

**Published:** 2026-06-12

**Authors:** Felicitas von Usslar, Büşra Ece Günaydın, Cordt Zollfrank

**Affiliations:** TUM Campus Straubing for Biotechnology and Sustainability, Technische Universität München, Straubing, Germany

**Keywords:** Biomaterials, Biomaterials

## Abstract

Cellulose fibrils are a renewable and biodegradable resource, but their extraction typically requires complete destruction of the original wooden matrix. We present a targeted strategy that enables selective liberation of cellulose microfibrils while preserving the integrity of the surrounding native wood structure. By combining partial delignification with localized surface modification using the ionic liquid 1-butyl-3-methylimidazolium acetate ([Bmim][OAc]), we enable selective liberation and separation of cellulose microfibrils without bulk dissolution or structural damage. This spatially confined treatment exploits the intrinsic anisotropy of the native cellulose architecture, allowing controlled fibril release while maintaining the original orientation and structural framework. These findings reveal how precise chemical interventions can expand the toolbox of cellulose chemistry and unlock new opportunities for advanced wood-based material engineering.

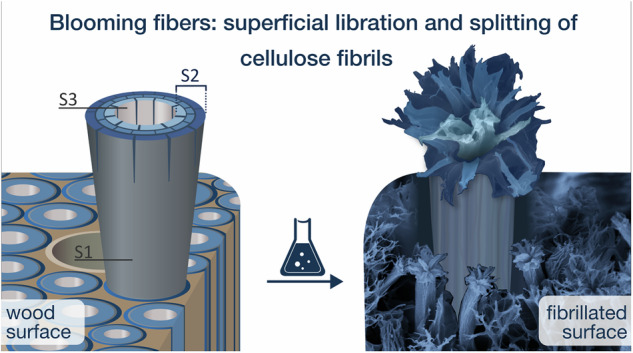

## Abstract (web summary)

Cellulose fibrils are a renewable and biodegradable resource, but their extraction typically requires complete destruction of the original wooden matrix. Here, the authors combine partial delignification with localized surface modification using 1-butyl-3-methylimidazolium acetate ([Bmim][OAc]) to achieve controlled fibril release while maintaining the original orientation and structural framework of the surrounding native wood structure.

## Introduction

In recent years, the polysaccharide cellulose has attracted growing interest in (bio)polymer research, not only due to its high natural abundance and renewability but also because of its remarkable versatility across a broad range of applications. Recent cellulose-related studies range from construction materials, such as thermal insulation^1,2^, to advanced bio-based composites^3^. Its biodegradability, combined with the capacity for adaptable structural and chemical modification, makes cellulose a key material in sustainable materials development^4,5^.

Among cellulose sources, the wood cell wall matrix is probably the most intensively studied, offering opportunities for innovations in waste valorization, pulp and fiber optimization, and bioinspired material design^6^. Concurrently, materials science increasingly draws inspiration from biological systems, leveraging the sophisticated structures and composite designs found in nature to solve complex engineering challenges. This shift in perspective reinforces the value of examining the native hierarchical multiscale architecture of wood as a functional template for sustainable advanced material systems^7^.

**Fig. 1:**
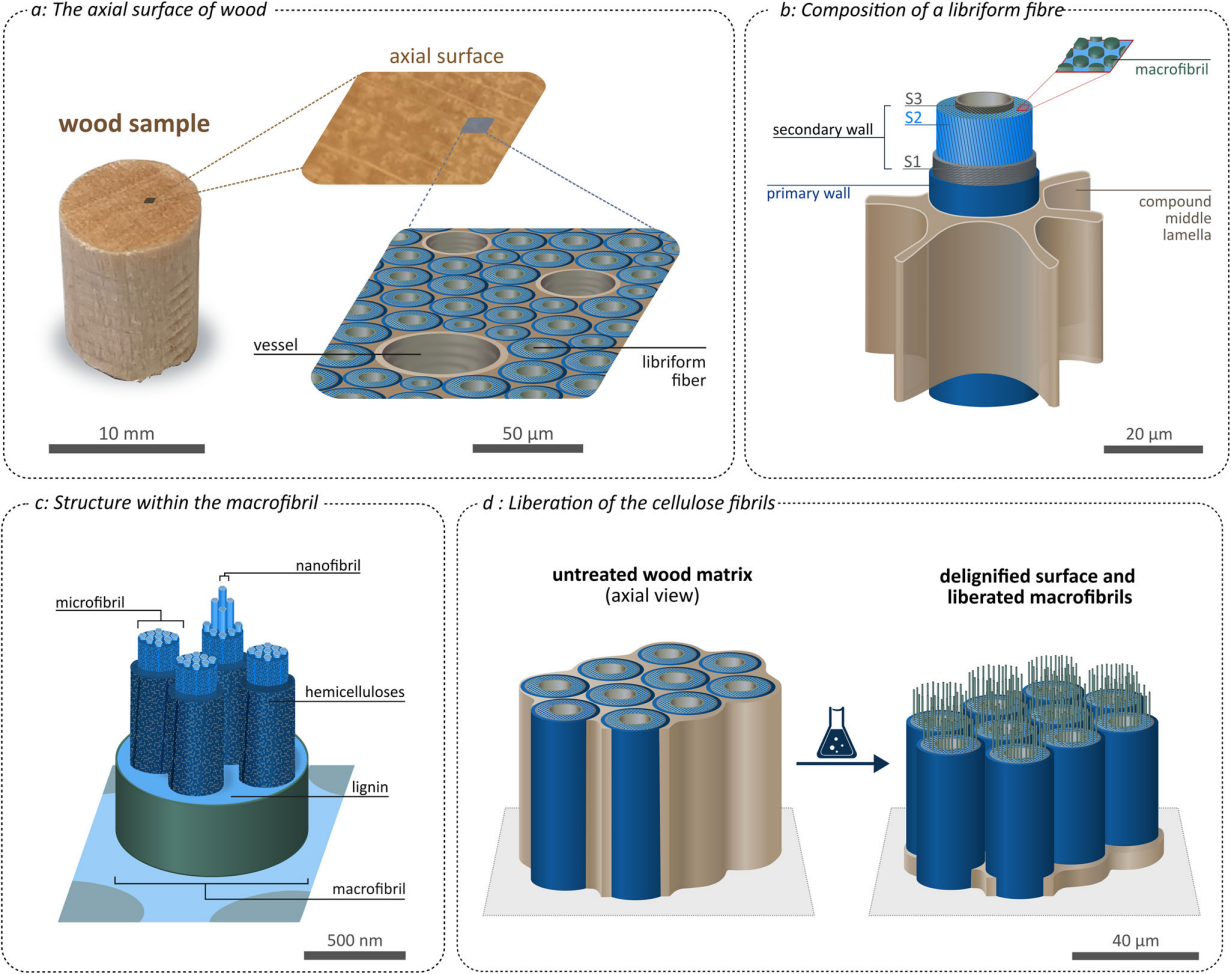
Wood composition and scope of this project. **a** The cross-section surface of a wood sample from which the cellulose fibrils are liberated. **b** Layered composition of a hardwood cell wall. Only in the S2 wall, the cellulose fibrils are in nearly upright position, it is composed of cellulose macrofibrils. **c** Hierarchical composition of a cellulose macrofibril. **d** Schematic liberation of cellulose fibrils from the axial wood matrix through chemical treatment. The remaining compound middle lamella symbolizes the remaining anchoring wood matrix.

Wood provides a naturally embedded, hierarchically organized cellulose fibrillar framework. Hardwood xylem is composed of vessels, fibers, and parenchyma cells (Fig. 1a), with libriform fibers being the primary contributors to mechanical strength. These fibers are elongated cells built from a hierarchical cell wall structure consisting of a primary cell wall (P) and secondary cell wall layers (S1, S2, and S3; Fig. 1b). At the nanoscale, cellulose fibrils assemble into microfibrils that are embedded in a matrix of hemicelluloses and lignin, forming a composite material^8–10^. The orientation of cellulose microfibrils varies across the secondary wall layers, with the S2 layer being the thickest, exhibiting microfibrils that are predominantly aligned with the cell axis (typically 5–30°), thereby playing a major role in determining the mechanical properties of the fiber^11–13^. Lignin is composed of three irregularly repeating monolignols: sinapyl, coniferyl, and coumaryl alcohol^14^, linking primarily to the hemicelluloses through ether bonds, occasionally also to cellulose (Fig. 1c). This molecular organization yields a hierarchically structured biopolymer composite combining the toughness and partial crystallinity of cellulose fibrils with the flexibility and chemical variability of hemicelluloses and lignin^15–17^.

In order to liberate the vastly integrated cellulose fibrils from the wood matrix, numerous strategies for (partial) delignification have been developed, permitting the fabrication of a wide range of advanced, wood-derived functional materials^18–21^. In particular, modification of the axial surfaces of libriform fibers has enabled the tuning of structural properties for specific applications. These modifications extend the functionality of wood-based materials beyond their native states, even when the cellulose fibrillar framework is not the principal focus^22^. Due to the hierarchical organization of wood (Fig. 1a–c), delignification facilitates the extraction of cellulose macrofibrils (Fig. 1d). While the removal of the lignin-rich middle lamella allows for complete separation of individual cells, the dissociation of elementary cellulose fibrils into discrete nanofibrils remains unachievable. This is generally achieved via rigorous mechanical disintegration of the bulk material, which always leads to full destruction of the wood matrix. This rules out any surface treatment while keeping the resulting fibers anchored in the wood matrix and thus securely situated in place according to their original framework^23–26^. From a bottom-up perspective, fabricating an aligned array of anchored cellulose fibrils perpendicular to the surface would require precise spatial control and further stabilization within a synthetic matrix. In contrast, our top-down approach targets the surface of a wood cross-section only, locally liberating cellulose nanofibrils while preserving their native alignment and their fixation to the wood matrix. This strategy leverages the intrinsic anisotropic alignment of cellulose within the wood’s natural architecture, but has not been realized experimentally. Moreover, it addresses key challenges in sub-micron structuring by retaining the ordered structural framework provided by native wood. Effective delignification requires cleavage of ether linkages connecting lignin and carbohydrate polymers, which otherwise anchor lignin within the cellulose–hemicellulose matrix^27,28^.

Here, we introduce a method that combines partial delignification of a wood cross-section with localized treatment using the ionic liquid (IL) 1-butyl-3-methylimidazolium acetate ([Bmim][OAc]) to selectively liberate cellulose fibrils still partially embedded in their original wood matrix. Unlike conventional processing, the IL does not dissolve cellulose but disrupts residual matrix components, promoting fibril separation while preserving the native orientation of cellulose in the tissue.

A surface of free-standing cellulose fibrils provides a larger surface compared to its original wooden or delignified state, thus providing a bio-based scaffolding, for example, for immobilizing catalysts, or additional biopolymers towards adhesive surfaces^29^. Further possible use cases could be ion exchange functionalities based on a biogenic platform using already established reactivities^30,31^.

The study shows that combining partial delignification with localized ionic-liquid treatment enables controlled, surface-restricted liberation of oriented cellulose microfibrils from wood without structural destruction, revealing hierarchical cell-wall disassembly and offering a route to engineered wood-based materials.

## Results and discussion

Building on the hierarchical structure of wood xylem described above, the internal architecture of libriform fibers is central to their chemical and structural behavior. The cell wall is organized into a thin primary wall and three secondary wall layers (S1, S2, and S3; Fig. 1b) separated by the lignin-free middle lamella. These layers differ in composition and accessibility, which is relevant for subsequent chemical treatment. In particular, hemicelluloses, while exhibiting a degradation resistance comparable to cellulose, are more susceptible to hydrolysis due to their lower crystallinity, reduced molecular weight, and the presence of less stable pentose units^16,32^.

### Locally confined delignification

**Fig. 2:**
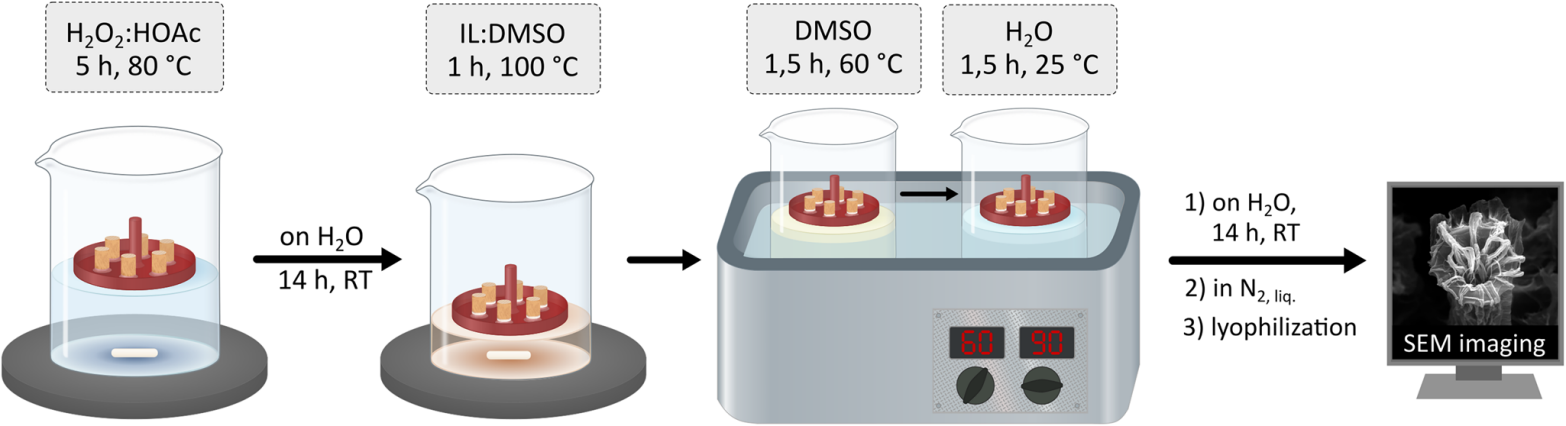
Schematic process of wood sample treatment, from delignification to lyophilization. Depicted is the floating custom sample holder being used during the different steps throughout the treatment process.

The penetration depth of the reaction solution can be limited to approximately 2 mm by employing a floating reaction system that seals sample sides and controls diffusion through a viscous delignification solution (Figs. 2, S2, and S3). In comparison to traditional delignification processes using NaClO_2_ or NaSO_3_, in situ formation of peracetic acid (PAA) from hydrogen peroxide and acetic acid is less toxic and more selective, leaving the cellulose favorably intact^33,34^. The addition of polyacrylic acid then provides an adjustable viscosity to the reaction solution, which is necessary to prevent the rapid infiltration of the entire wood body due to capillary forces. Controlled polymer degradation ensures recyclability and maintains high surface viscosity throughout the reaction. The partially delignified samples are washed and subsequently treated with an IL:dimethylsulfoxid (DMSO) mixture using the same floating configuration. The floating method ensures locally restricted contact to the pretreated axial wood surface. Although capillary uptake caused visible darkening via limited infiltration through large vessels, this effect was negligible and fully reversible after washing.

A systematic study was conducted to identify the optimal conditions for ionic liquid (IL)-based treatment of cellulose fibers, with the primary objective of promoting fibrillation of fiber ends.

Initial experiments focused on the IL 1-ethyl-3-methylimidazolium acetate (EmimOAc) in combination with dimethyl sulfoxide (DMSO) as a co-solvent. Mixtures of EmimOAc/DMSO at volumetric ratios of 1:1 and 1:2 were evaluated under varying temperature and reaction time conditions. It was observed that shorter reaction times (1 h) combined with elevated temperature (100 °C) resulted in improved fiber end opening compared to longer reaction times (3 h) at lower temperature (80 °C). Increasing the DMSO fraction alone did not improve outcomes under low temperature and prolonged reaction conditions. Even when high temperature was combined with extended reaction time, the system predominantly yielded a hydrogel-like matrix before freeze-drying, and porous aerogel in the dried state, indicating excessive dissolution and subsequent regeneration rather than controlled fibrillation. The effect of additives was investigated by introducing inorganic salts. The addition of LiCl promoted partial separation and opening of fiber ends, suggesting enhanced disruption of intermolecular interactions within the cellulose structure. In contrast, NaCl showed no measurable effect under comparable conditions, indicating that the observed improvements are specific to the lithium cation and its interaction with the IL–cellulose system.

A second ionic liquid, 1-butyl-3-methylimidazolium acetate (BmimOAc), was evaluated under similar conditions. At a 1:2 IL/DMSO ratio, low temperature, and extended reaction time, the system resulted predominantly in the formation of a gel-matrix on the surface. The addition of LiCl in combination with a short reaction in elevated temperature produced significantly improved fibrillation and fiber end opening. Control experiments with NaCl again showed no effect, confirming the specificity of LiCl in facilitating the desired morphological changes. Based on these observations, the optimal treatment conditions were identified as BmimOAc in a 1:2 ratio with DMSO, combined with LiCl as an additive, at 100 °C for 1 h.

Post-treatment processing involved a two-step washing procedure to remove residual IL and solvent. First, samples were washed in DMSO using an ultrasonic bath to extract ionic liquid residues. This was followed by washing in water to fully remove DMSO. The use of ultrasonication was confirmed not to induce fibrillation, as verified by control experiments (see Fig. S4 in the Supporting Information). Finally, the cleaned samples were subjected to freeze-drying to obtain and preserve the fully fibrillated structure (see Fig. S5 for the full process).

A mechanistic hypothesis was developed to explain the observations: prolonged reaction times promote partial dissolution of cellulose within the IL/DMSO system, forming a gel-like intermediate. Upon solvent exchange during washing (DMSO followed by water), this gel regenerates into a hydrogel, which subsequently collapses during freeze-drying into a thin aerogel or foam-like layer. This pathway is consistent with the formation of a non-fibrillated porous matrix observed under conditions involving extended reaction times. In contrast, shorter reaction times at elevated temperatures favor controlled disruption of fiber interfaces without complete dissolution, enabling fibrillation rather than bulk gel formation. Supporting images and data are provided in the Supporting Information (Table S1 and Fig. S6).

### Selective fibrillation/splitting fibers

Imidazolium-based ionic liquids (ILs) are a class of high-potential solvents, which exhibit high thermal stability due to strong ionic binding, permitting elevated reaction temperatures without solvent power loss^35^. Mechanistic studies have shown that the anions primarily govern the dissolution of lignin and cellulose in ILs, such as [Bmim][OAc], whereas the cation is the dominant factor for the separation of cellulose and lignin^36^. Accordingly, such ILs are promising candidates for pretreatment processes aimed at accessing the cellulose fibrils in bulk material, followed by full matrix dissolution using enzymes^37^. DMSO was chosen as a cosolvent to improve the mass transport and accelerate fibrillation reaction through the modulation of viscosity. While DMSO is known to induce physical changes in cellulose, including swelling and increased accessibility of the fiber structure, literature reports indicate that it does not significantly affect the intrinsic interaction between [Bmim][OAc] and cellulose^38–40^. Its influence is therefore mainly exerted through changes in transport properties and cellulose morphology, which can affect dissolution and fibrillation kinetics without altering the underlying interaction mechanism. The addition of a small amount of LiCl markedly improved fibrillation outcomes, likely due to chloride acting as a smaller, more reactive anion. Li⁺ cations can coordinate with solvent molecules and insert into cellulose hydrogen-bond networks, enhancing their separation^41,42^. We propose that the chloride acts as an adjuvant anion, reaching additional reaction sites at the cellulose and hemicelluloses due to its smaller size compared to the acetate anion. This is supported by the findings of Xu and coworkers, who reported the higher solubility of IL/LiCl systems to be explained by the Li^+^ cation inserting itself into the inter-molecular hydrogen bonds of cellulose, forcing their separation^43^.

**Fig. 3:**
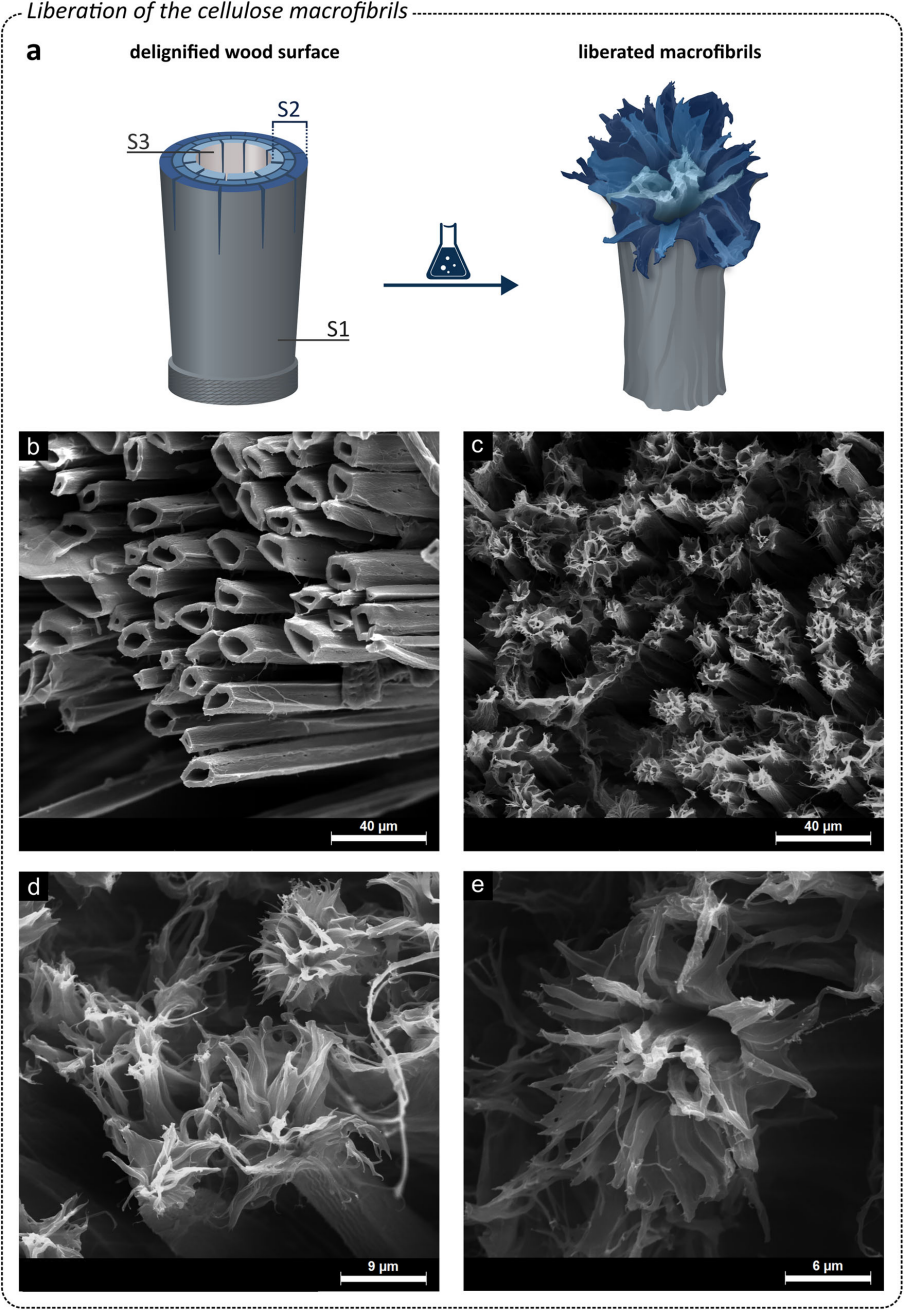
Change in surface morphology before and after IL-treatment. **a** Scheme of a libriform fiber before (left) and after liberation of cellulose macrofibrils (right). **b** SEM image of delignified libriform fibers of basswood before treatment, corresponding to a (left). **c**–**e** SEM images of partly liberated cellulose fibrils on the axial surface of libriform fibers of basswood after treatment corresponding to a (right).

FTIR-ATR spectroscopy confirmed extensive delignification (Fig. S1 and Supplementary Data 1). Imaging by scanning electron microscopy (SEM) revealed that IL treatment with [Bmim][OAc]:LiCl produced distinct fibrillation within the first 10 micrometers (µm) of the libriform fiber surface of basswood (Fig. 3). The S2 layer frequently exhibited concentric delamination, consistent with reported swelling perpendicular to lamellae under wet conditions (see also Fig. S7)^44,45^. We can therefore assume that the wet and thus swollen delignified matrix provides accessible reaction sites for the IL, which then intercalates between hemicelluloses, lignin residues, and eventually between cellulose microfibrils. Images acquired by SEM show extensive fibrillation across the axial surface, typically forming macrofibrils 100–500 nm in diameter with a depth of 6–10 µm (see also Fig. S8).

**Fig. 4:**
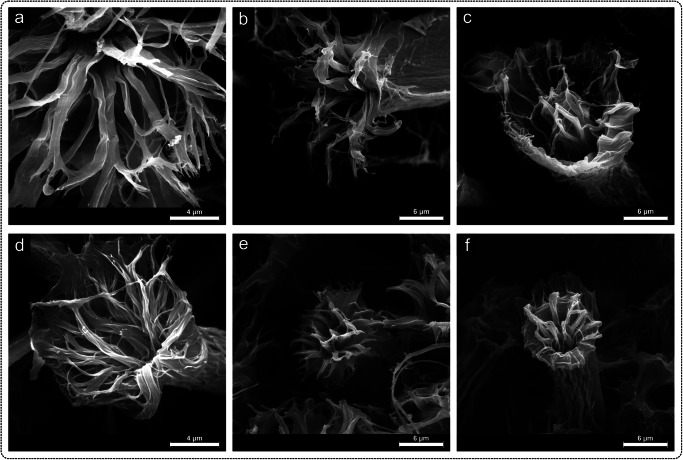
SEM images of three observed differing patterns of fibrillation in libriform fibers after IL-treatment. **a**, **d** Hierarchical splitting. **b**, **e** Macrofibril-ribbons with some visible microfibrillation. **c**, **f** Fibrillation within the S2 cell wall, leaving the S1 cell wall intact.

**Fig. 5:**
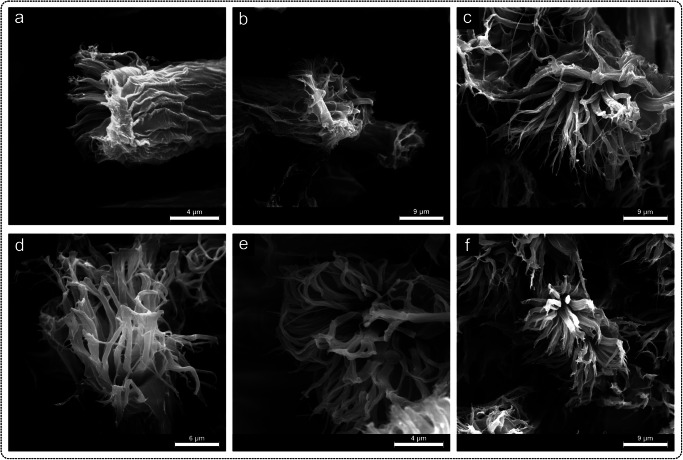
SEM images of basswood libriform fibers treated with [Bmim][OAc]. **a**, **b** Side view of treated basswood fibers showing the contracted outer surface. **c**, **d** Liberated macrofibrils splitting down into microfibrils (⌀ = 200 nm). **e**, **f** Liberated macrofibrils clustered in bundles at the tips despite prior separation.

Three dominant fibrillation morphologies emerged: (i) hierarchical splitting (Fig. 4a, d); (ii) ribbon-like delamination ending in discrete macrofibrils (Fig. 4b, e); and (iii) localized fibrillation within the S2 region bound to an intact S1 layer (Fig. 4c, f). Fibrillated fibers (Figs. 3e and 4a–f) display concentric delamination of the S2 cell wall. However, often the first part of the secondary wall (S1) seems to stay intact longest (Figs. 4c, f, and 5a–c). We assume that these three morphologies represent three different states of reaction progress with the ionic liquid, depending on the dimensions, local environment, and conditions of each singular libriform fiber in the original array (see also Fig. S9).

The exterior of the cellulose fibers changes its morphology as well. The fibers show surface contraction in relation to delignified-only samples after the treatment (Fig. 5a, b), suggesting internal removal of some material, i.e., the lignin and hemicelluloses between the cellulose fibrils^34^. A film-like barrier limits fibril separation here, and fine striations on the exterior support our interpretation that this is the S1 layer of the secondary cell wall^46,47^.

Unlike the IL-induced partial dissolution observed by others using a guanidinium phosphorus-based IL-DMSO mixture^48^, our results indicate a different mechanism here, based on the different stages of formation observed. From a structural point of view, the more readily accessible S2 wall is axially infused by the IL, resulting in lamellar separation in the magnitude of cellulose macrofibril sheets. The combination of short reaction time, high temperature, and ultrasonic washing was essential for effective fibrillation, which is maintained through cryofixation and lyophilization. Lower temperatures, altered durations, or fully dried samples yielded negligible results, highlighting the importance of matrix pre-swelling for IL efficacy.

## Conclusions

Surface-restricted fibrillation was ultimately achieved via selective delamination of the cellulose/hemicellulose-lignin matrix in the S2 wall of basswood libriform fibers. Partial delignification facilitated macro- and microfibril separation, which was preserved through cryofixation and lyophilization. Rapid ice crystal growth spread fibril bundles apart, producing “blooming” released cellulose fibril ends and revealing the hierarchical disassembly of the cell wall down to the microfibril scale.

This study demonstrates that ILs can locally liberate cellulose structures within wood below the ubiquitous libriform fibers without mechanical disruption, generating an anisotropic oriented cellulose fibril-terminated surface that remains anchored to the original wood matrix. The approach provides a pathway for species-specific surface engineering of wood-based materials. Given the diversity of wood species and structural architectures, further exploration of IL-wood interactions may unlock new strategies for tailored, sustainable cellulose-based materials.

## Materials and methods

### Partial delignification and ionic liquid treatment

Selective delignification was performed according to Gürer et al., with minor changes^29^. The wood (basswood, *Tilia spp*.) was acquired from a local supplier. Cut into cylindrical pieces (*⌀* 8 mm × 10 mm), the wood samples were subject to extraction with H_2_O/acetone (4:1 v/v, 115 °C, 24 h) and toluene (140 °C, 24 h; ≥99.5%, Carl Roth). The axial surface of each sample was then cut using a microtome (Reichert–Jung Ultracut E) to ensure an even, planar surface. For surface confined delignification, the wood samples were floated in a custom sample holder from silicone rubber (1:1 w/w; TFC Silikon Kautschuk Typ 3 HB, Troll Factory, Riede, Germany; see also Fig. S2) on a solution of hydrogen peroxide (H_2_O_2_, 35%, pure, stabilized, Carl Roth) and glacial acetic acid (1:1 v/v; 100% purity, VWR, Radnor, PA, USA) with addition of 2 wt% Carbopol 971P NF (Lubrizol, Wickliffe, OH, USA) for 6 h at 80 °C. After washing the samples by floating on 800 ml H_2_O for 12 h at room temperature, the still damp samples were treated with [Bmim][OAc] (BLD Pharmatech GmbH, Reinbeck, Germany) and DMSO (1:2 v/v; ≥99.97%, Sigma-Aldrich, St. Louis, MO, USA) with the addition of LiCl (7–13 mmol eq.; VWR) for 1 h at 100 °C. The subsequent floating washing process was done in an ultrasonic bath (60 °C) in DMSO (1.5 h) and then H_2_O (1.5 h), followed by floating on 800 ml H_2_O at room temperature overnight. For drying, the samples were frozen by dipping them with the treated side first in liquid nitrogen and then lyophilized for a minimum of 4 h (−45 °C, 0.080 mbar, Christ Alpha 2-4 LDplus, Christ, Osterode, Germany).

### FTIR-ATR

To verify the removal of lignin, Fourier-transformed infrared spectroscopy (FTIR) was performed on the dried sample surface in attenuated reflection mode (ATR) with 16 scans at a resolution of 2 cm^−1^ (4000–550 cm^−1^, Frontier, Perkin Elmer, Waltham, USA). Apart from the visual difference in color, missing modes of aromatic skeletal vibration (1508 cm^−1^) and C–H-bending (1460 cm^−1^) of lignin point toward successful removal of lignin and lignin fragments at the treated sample surface. This is supported by increased intensity of modes assigned to O–H in-plane bending (1333, 1315, and 1205 cm^−1^) and C3–O3H-stretching (1050 cm^−1^) of cellulose and hemicelluloses^49–51^. See Fig. S1 and Supplementary Data File 1.

### Scanning electron microscopy

For detailed imaging with the scanning electron microscope, the sample surface was carefully cut, mounted with carbon pads, and sputter-coated (Pd/Au, 90 s at 40 mA, Bal-Tec SCD 050, Balzers, Liechtenstein). Images were acquired at 20 kV, detecting secondary electrons (DSM 940A, Zeiss, Germany).

## Supplementary information

Supplementary Information

Description of Additional Supplementary Files

Supplementary Data 1

## Data Availability

The authors declare that the main data supporting the findings of this study are available within the article and its Supplementary Materials.
