# Supplementary material for: Selective cellulose fibril release from hardwoods libriform tissue
Source: Commun Chem. 2026 Jun 12;9:203. doi: 10.1038/s42004-026-02094-4 (PMC13263335; doi:10.1038/s42004-026-02094-4)
Supplement: Supplementary file 1 — Supplementary Information [file 42004_2026_2094_MOESM1_ESM.pdf]

Supplementary Materials for

Selective cellulose fibril release from hardwoods libriform tissue

Felicitas von Usslar, Büsra Ece Günaydın, Cordt Zollfrank

Corresponding author: cordt.zollfrank@tum.de

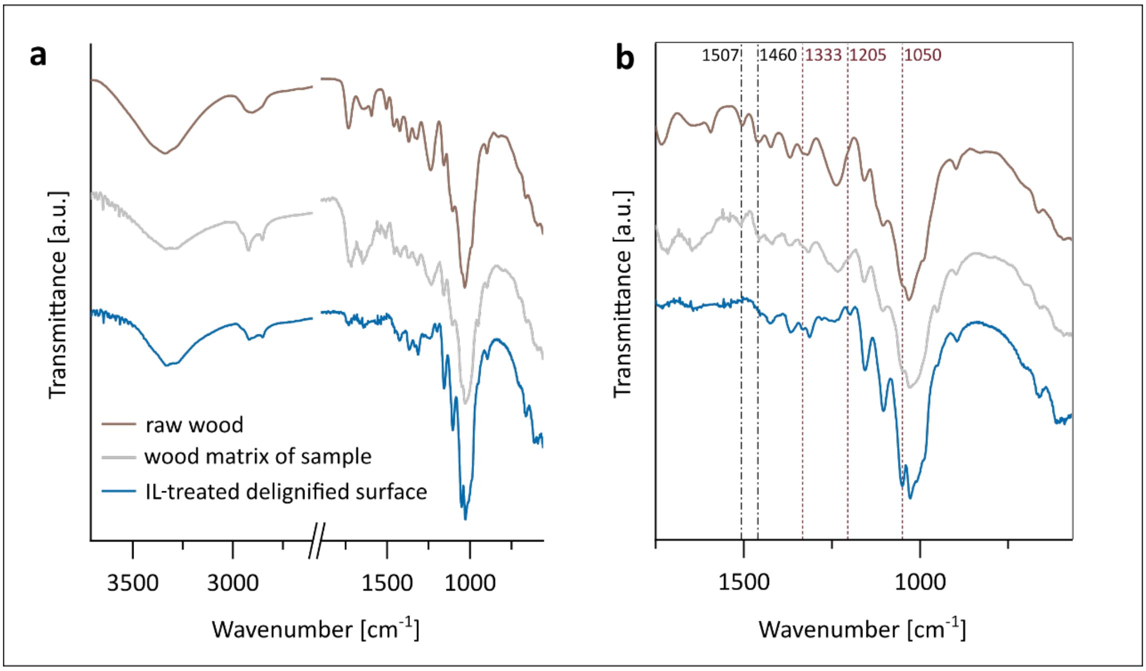

Fig. S1: FTIR-ATR spectra of raw and treated wood samples.

a: FTIR spectra of untreated wood, the wooden end of the sample after treatment and of the delignified end of the treated sample; b: Close-up of the modes of interest, mentioned in the methods section. Numerical data is provided in the file Supplementary Data 1.

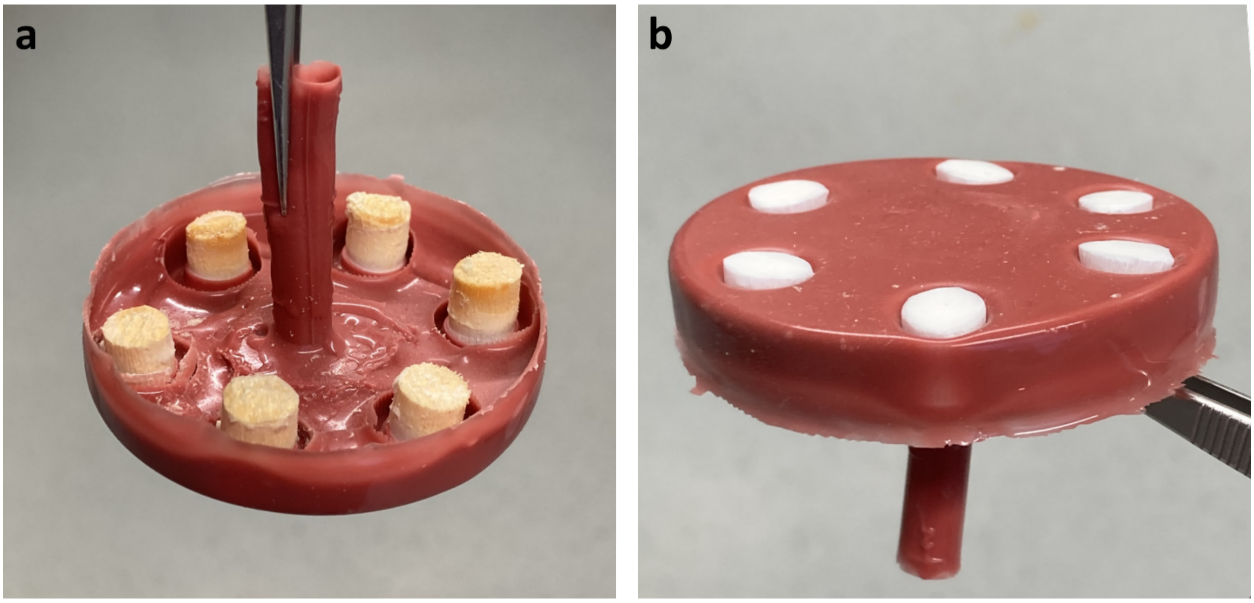

Fig. S2: The floating sample system used for the treatment of wood samples.

The silicone sample holder was constructed as described by Gürer et al. (43). Holder for two to eight samples were constructed with a diameter of 6 cm. Here shown with samples after the delignification process; a: View upright; b: View from the bottom.

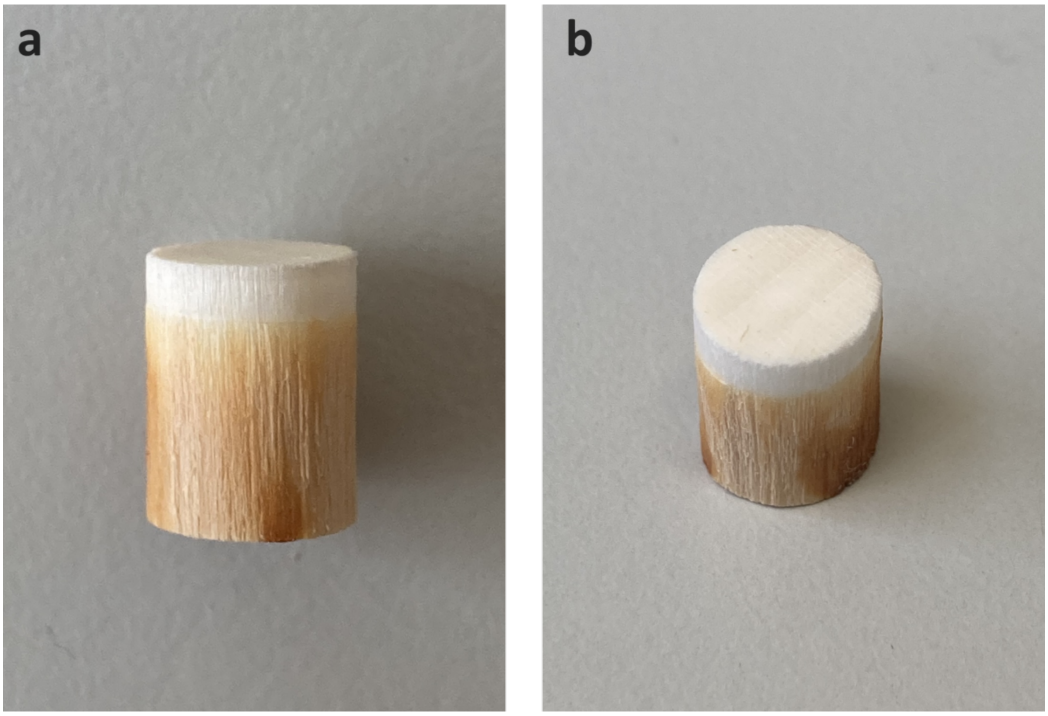

Fig. S3: Photographs of a partially delignified wood sample (basswood).
a: View from the side (longitudinal) and b: View of the axial surface. The diameter of the wood cylinder is 8 mm.

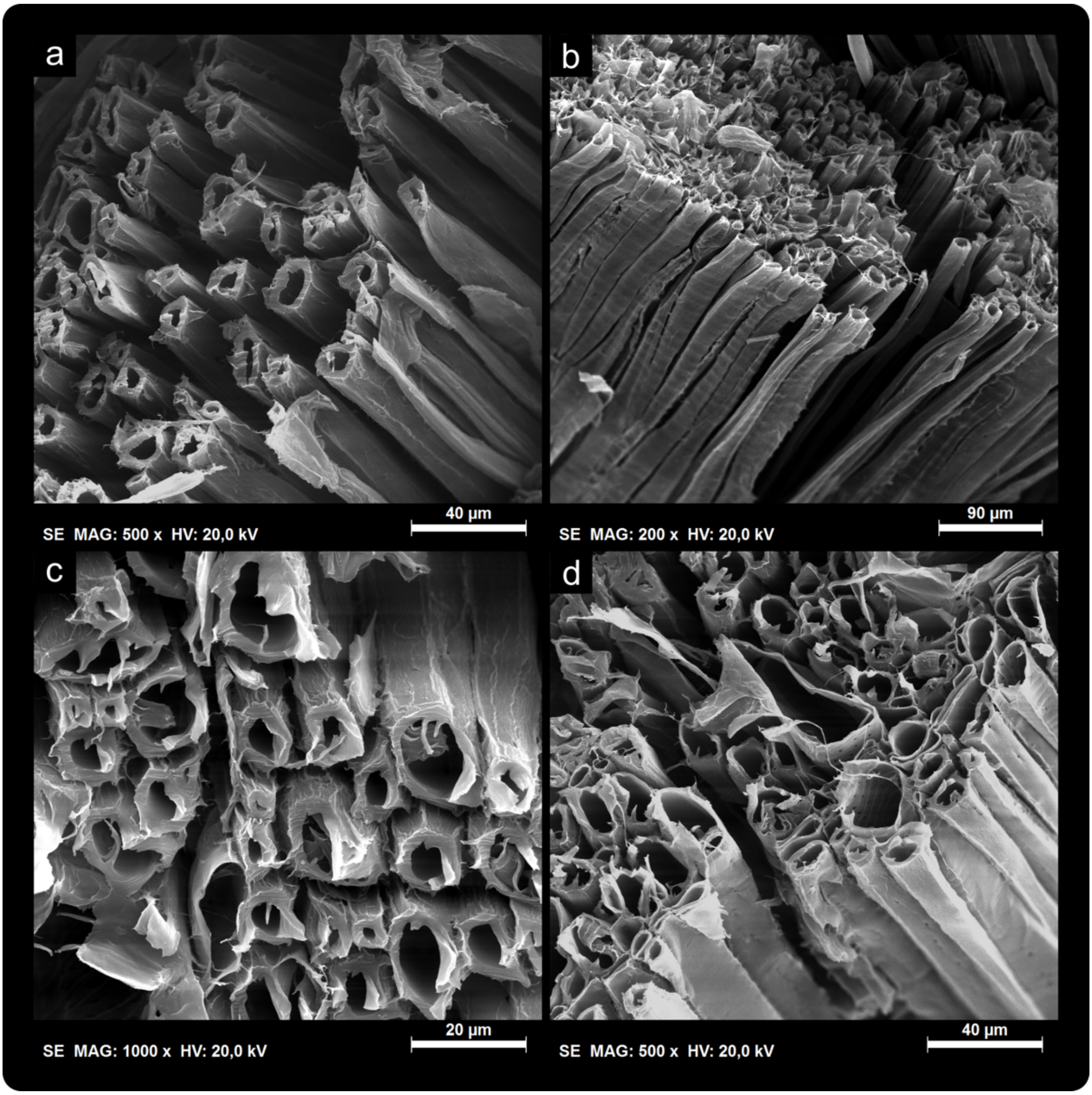

Fig. S4: SEM pictures of delignified and IL-treated basswood surface (axial).

a, b: Images of a basswood surface after treatment as described above, washed by only floating on 800 ml water over night; c, d: Images of a basswood surface after treatment with EmimOAc: DMSO (1:1, v/v) for 1 h at 60 °C, subsequently washed using the ultrasonic bath washing process described above.

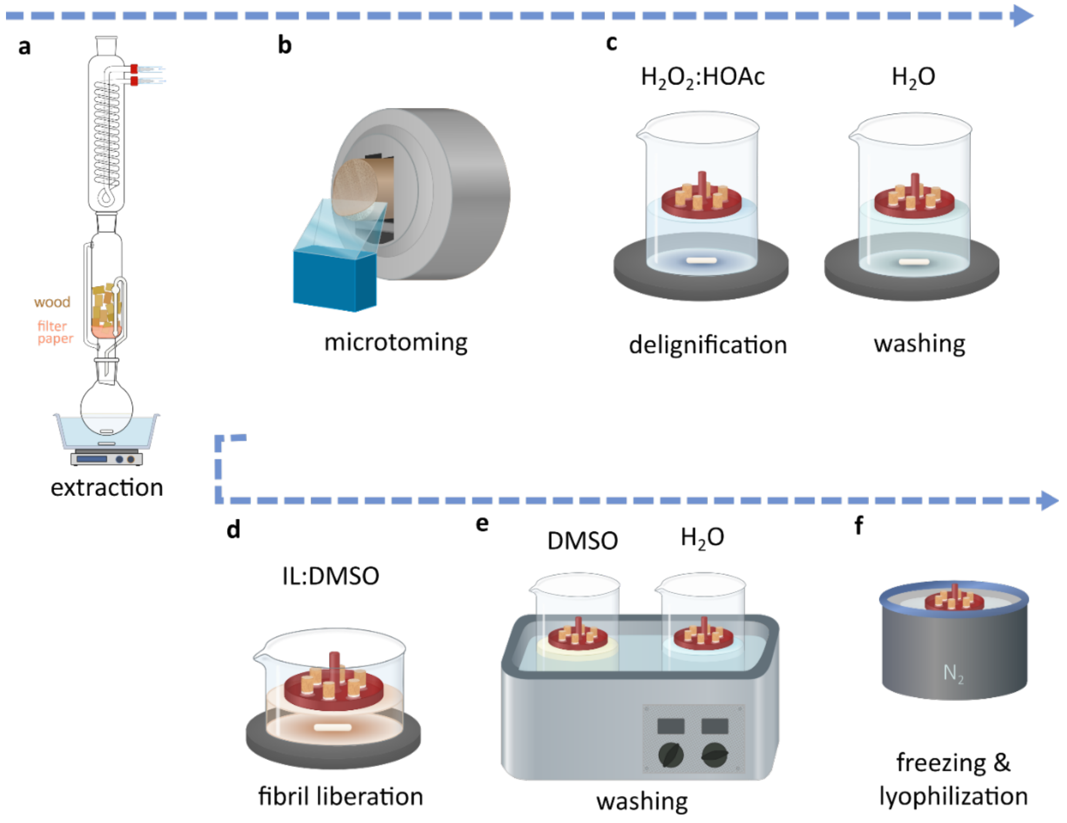

Fig. S5: Illustration of the whole process for fibrillation described above.
a: Extraction of the wood; b: Planning the surface to ensure a level axial surface; c: Delignification followed by the floating washing process; d: Treatment with ionic liquid in DMSO; e: Floating washing process in ultrasonic bath; f: Cryofixation in liquid nitrogen followed by lyophilization.

Table S1: Overview of reaction conditions and their results.
For reference images for different surfaces see Figure S6.

Ionic Liquid	Solvent	Ratio	$T_{reaction}$ [°C]	$t_{reaction}$ [min]	Salt	Result
EmimOAc	DMSO	1:1	80	180	--	some opening of fiber ends
		1:1	100	60	--	opening of fiber ends *(S6a,b)*
		1:2	80	180	--	porous matrix *(S6c,d)*
		1:2	100	60	--	opening of fiber ends
		1:2	100	180	--	porous matrix
		1:3	100	60	NaCl	no change *(S6g)*
		1:2	100	60	LiCl	some delamination
BmimOAc	DMSO	1:1	75	60	--	no change
		1:1	75	300	--	delamination
		2:3	70	270	--	delamination *(S6e)*
		1:2	80	180	--	porous matrix *(S6f)*
		1:2	100	60	LiCl	fibrillation
		1:2	100	60	NaCl	control, no change *(S6h)*

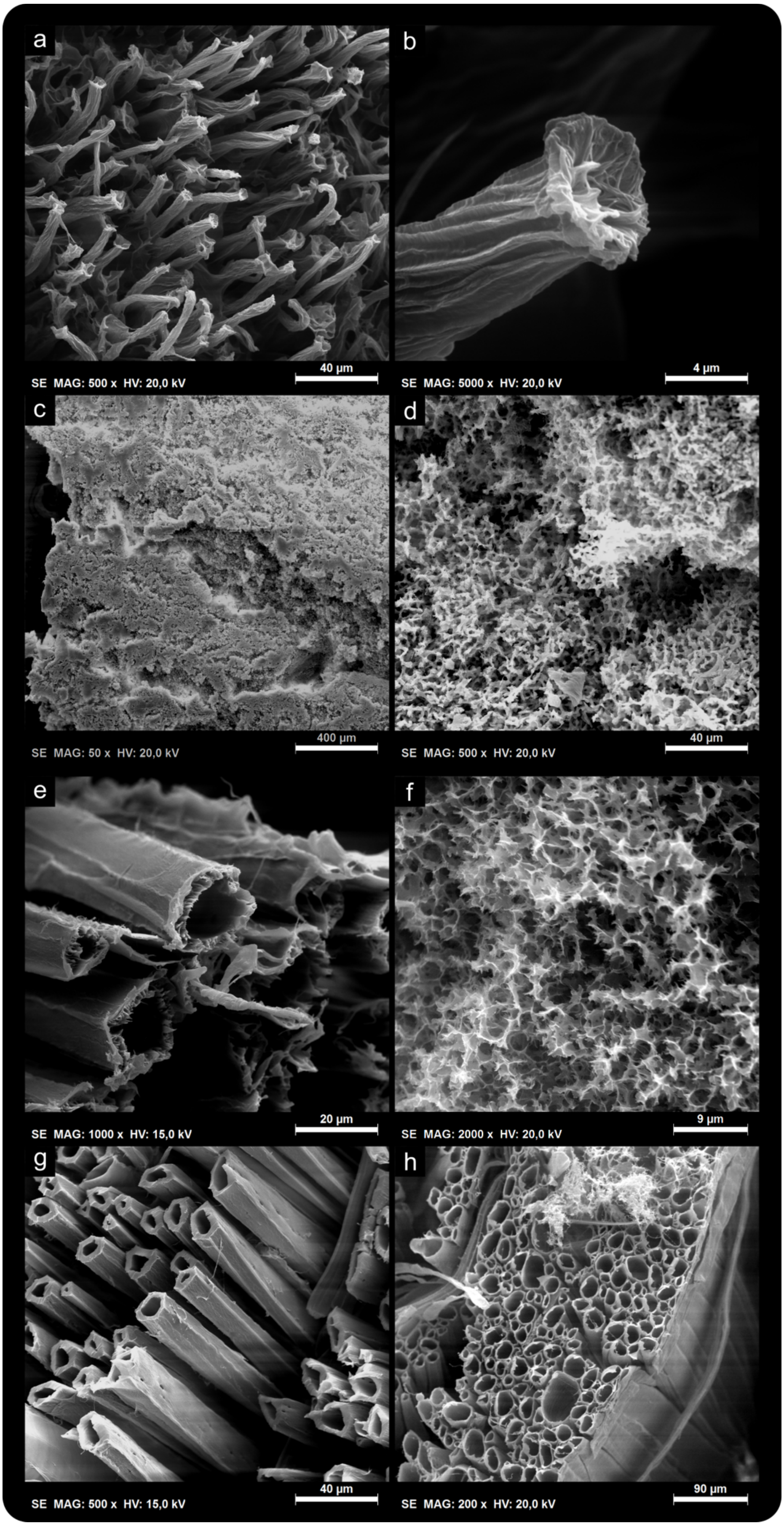

Fig. S6: SEM images of resulting surfaces after IL treatment, as referenced in Table S1.

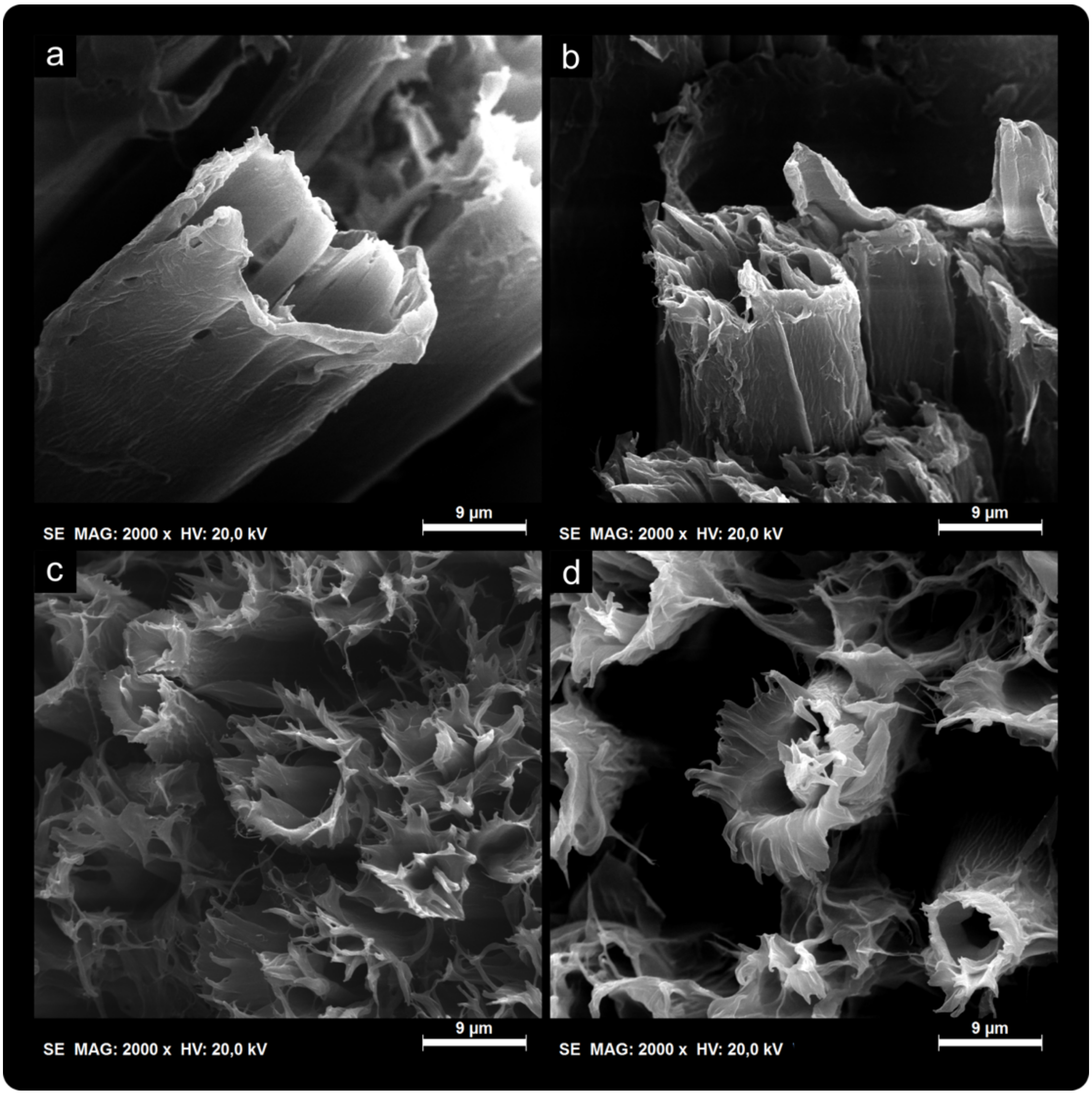

Fig. S7: SEM images of delamination of fibers as pre-fibrillation state.
a, b: Some delamination can be seen on the inside of the fiber end, while the outer layer seems intact, yet (BmimOAc, DMSO (1:1 v/v), 75 °C, 5 h); c, d: Further advanced state of delamination, several layers can be distinguished and beginning of partial splitting can be seen (BmimOAc, DMSO (1:2 v/v), 100 °C, 1 h).

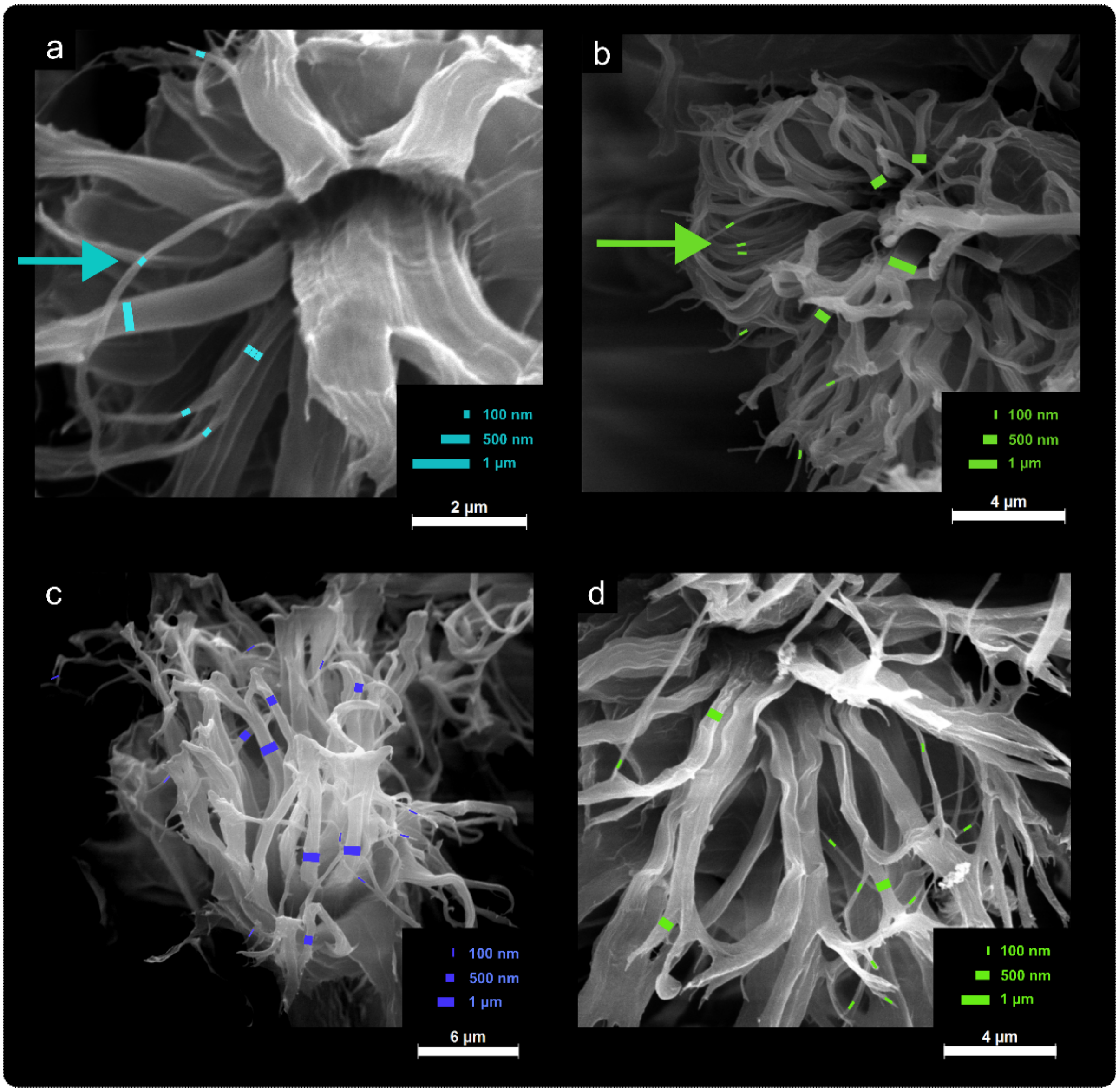

Fig. 8: SEM pictures of fibrillated cellulose at different magnifications.

To illustrate the fibrillation of the matrix down to microfibrils (below 200 nm in diameter), colored scale bars were added to the SEM images.

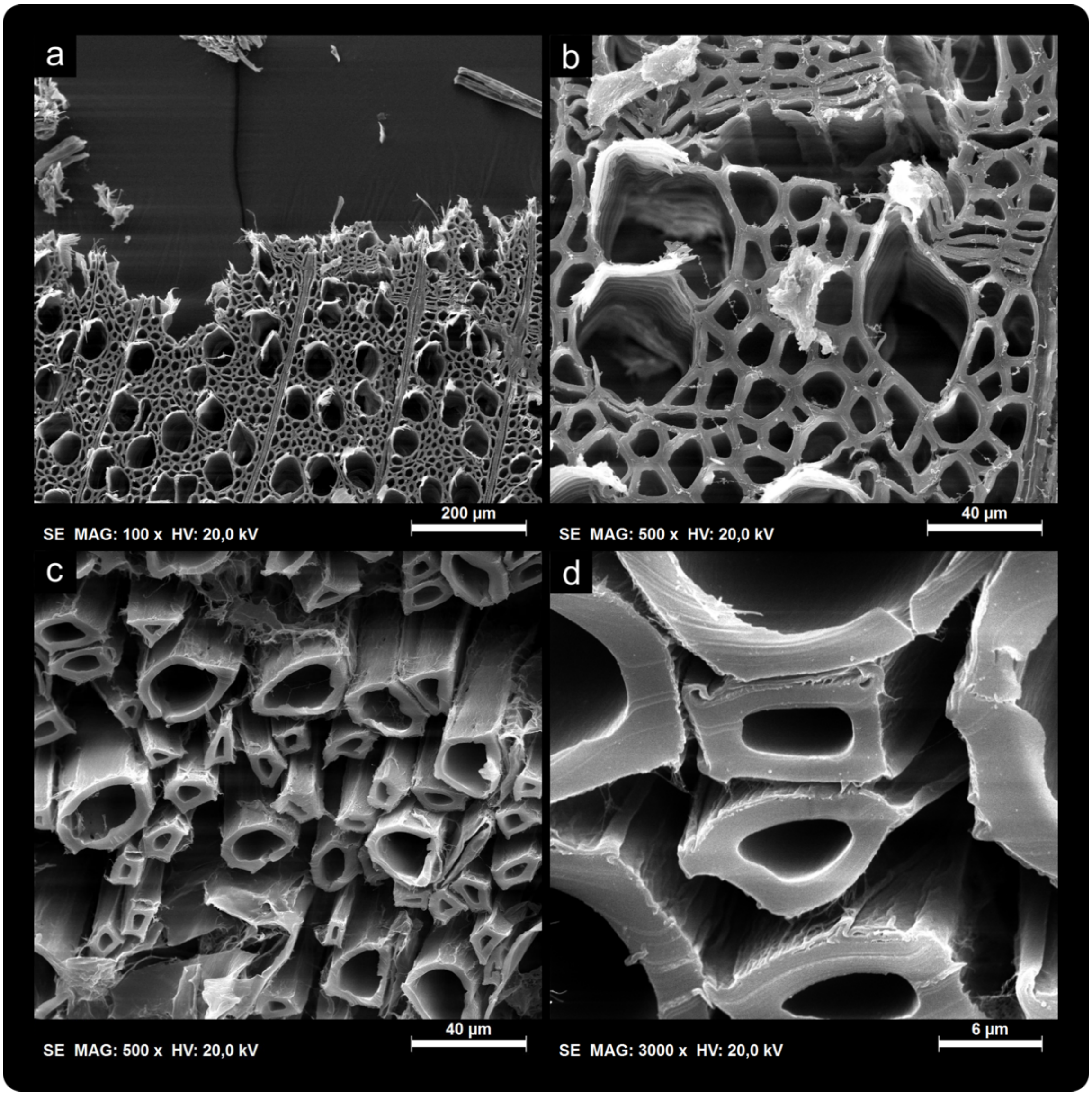

Fig. S9: SEM images basswood pre- and post-delignification

a, b: Axial cut of a basswood sample which was only subject to extraction; c, d: SEM images of the axial plane of delignified basswood. Following the removal of the compound middle lamella, the fibers stay intact but are separated from each other.
