# Supplementary material for: Selective cellulose fibril release from hardwoods libriform tissue
Source: Commun Chem. 2026 Jun 12;9:203. doi: 10.1038/s42004-026-02094-4 (PMC13263335; doi:10.1038/s42004-026-02094-4)
Supplement: Supplementary file 2 — Description of Additional Supplementary Files [file 42004_2026_2094_MOESM2_ESM.pdf]

Description of Additional Supplementary Files:

File name: Supplementary Data 1

Description: Source data for FTIR-ATR graph is given in the supplementary information.
